# Characteristics of media-reported road traffic crashes involving low-level automated vehicles in China, 2015–2025

**DOI:** 10.7189/jogh.16.04245

**Published:** 2026-08-28

**Authors:** Min Zhao, Lei Yang, Hengjia Qi, David C Schwebel, Wangxin Xiao, Yu Wu, Peixia Cheng, Guoqing Hu

**Affiliations:** 1 Department of Epidemiology and Health Statistics, Hunan Provincial Key Laboratory of Clinical Epidemiology, Xiangya School of Public Health, Central South University, Changsha, China; 2 Office of the Vice President for Research, University of Iowa, Iowa City, Iowa, USA; 3 School of Intelligent Biomedical Engineering, Hunan University of Technology and Business, Changsha, China; 4 Department of Maternal and Child Health, School of Public Health, Capital Medical University, Beijing, China; 5 Beijing Key Laboratory of Environment and Aging, Capital Medical University, Beijing, China; 6 National Clinical Research Center for Geriatric Disorders, Xiangya Hospital, Central South University, Changsha, China; 7 Laboratory of Intelligent Governance for Complex Social Systems, Changsha, China

**Keywords:** road traffic crash, low-level automated vehicles, media, China, injury

## Abstract

**Background:**

Low-level automated vehicles (LAVs) have emerged as a new public health challenge, but the epidemiological characteristics of LAV-related crashes remain unknown.

**Methods:**

Based on media reports collected by the Automated Road Traffic Crash Data Platform (ARTCDP), we analysed the characteristics of LAV-related crashes in China between 1 January 2015 and 31 August 2025.

**Results:**

The ARTCDP captured 4,669 crashes involving LAVs and 324,869 involving other motor vehicles. Compared to other motor vehicles, LAVs were more frequently reported to crash during nighttime (65.3% *vs*. 29.1%; *P* < 0.001), on expressways (31.9% *vs*. 20.2%; *P* < 0.001), on straight roads without junctions (50.7% *vs*. 29.4%; *P* < 0.001), and on rainy days (57.7% *vs*. 53.6%; *P* < 0.001). They were primarily reported to crash in economically developed regions, with those in Zhejiang, Guangdong, and Shanghai accounting for 31.6% of all crashes involving LAVs. Furthermore, 20.3% of the LAVs crashes and 16.3% of other motor vehicles crashes were associated with two or more factors. Brake-related issues (48.0% *vs*. 26.1%; *P* < 0.01), hazardous road surface condition (55.6% *vs*. 47.9%; *P* < 0.01), and distracted driving behaviour (28.7% *vs*. 9.8%; *P* < 0.01) more frequently occurred in LAV-involved crashes.

**Conclusions:**

Internet-based media reports detected distinct characteristics of road traffic crashes involving LAVs, meriting the attention of policymakers and law enforcement.

Automated vehicles, defined as vehicles that can operate without direct input from a driver in at least one critical safety control function such as steering, accelerating, or braking, are expected to offer significant road safety advantages by eliminating driver errors [1]. They are classified by the Society of Automotive Engineers into six levels, ranging from L0 (no driving automation) to L5 (full driving automation) [2]. In 2025, the primary use of automated vehicles on public roads in China involved low-level automated vehicles (LAVs) equipped with L1 and L2 driver support systems, while vehicles with L3 or higher levels of automation had limited, primarily pilot-level deployment only. Chinese sales of LAVs with L1 and L2 driver support systems increased from 8 million in 2020 to 20.4 million in 2024 [3], and they came to account for 68% of the vehicle fleet on Chinese roads as of December 2025 [4].

As the number of LAVs increases, road traffic crashes involving LAVs have emerged as a new challenge for road traffic injury prevention. Data on LAV-related crashes are scarce in nearly all countries, including China, since current road injury or fatality data surveillance systems do not include a specific variable indicating whether a vehicle was equipped with low L1 and L2 driver support systems [5]. Currently, the best available data for assessing the safety performance of LAVs are those reported by pre-trained drivers and manufacturers during LAV testing or field operation. Such reports suggest that compared to other motor vehicles, LAVs do not perform better in reducing road traffic injuries as they were expected to [6] and are more likely to be associated with severe crashes [7], with crashes involving LAVs primarily being caused by system failure (52%), driver-responsible automated vehicle disengagement initiations (30%), and external conditions such as road infrastructures and weather (11%) [8]. No published studies systematically compared the characteristics of road traffic crashes involving LAVs *vs*. those involving other motor vehicles.

Media news reports about traffic crashes offer a timely and freely available data source on crashes involving LAVs and could supplement police-reported and other road traffic crash information systems [9]. Such reports, however, must be considered cautiously, as journalists tend to preferentially cover more severe or high-profile crashes, which consequently results in selection bias within research based on this data [10].

Using media data from the Automated Road Traffic Crash Data Platform (ARTCDP), a data platform that automatically collects, summarises, and extracts key information of media reports from online Chinese media concerning road traffic crashes every 24 hours [11], we examined the characteristics of road traffic crashes reported in Chinese media sources between January 2015 and August 2025 involving LAVs and compared them with crashes involving other motor vehicles.

## METHODS

Media sources indexed by the ARTCDP, our data source, include 37,073 news and government websites and social media accounts geographically spanning across 31 Chinese provinces and over 98% of prefecture-level divisions in mainland China [11].

After obtaining the textual reports, we used DeepSeek to extract information concerning 27 structured variables that characterise basic characteristics of the crashes (date, location, non-fatal injuries and fatalities) and factors causing the crash (human factor, vehicle factor, road factor, and other factors) [11]. The ARTCDP captured 329,538 media-reported road traffic crashes between January 2015 (the month of the first LAV-related crash reported in the ARTCDP) and August 2025.

We defined crashes involving LAVs as those involving vehicles equipped with L1 or L2 automated driving capacities where drivers had unlocked and used relevant autonomous driving functions when the crash happened. The LAVs were identified from media reports through manual search according to key words, conducted by one trained researcher and independently verified by another (Table S1 in the **Online Supplementary Document**). Crashes involving ‘other motor vehicles’ were defined as all other motor vehicle crashes at the time of the crash. We did not consider reports on vehicles with L3 or higher levels of automation, as they had not been deployed beyond pilot tests in China during the study period.

The ARTCDP platform automatically removed duplicate media stories that reported the same road traffic crashes using an internal algorithm based on the reported crash time and location [11]. After keyword screening, a second deduplication was conducted by using the unique report IDs in Microsoft Excel. To assess relevance of the identified reports, we randomly sampled 10% of retrieved news reports for manual evaluation, and over 96% were found to be eligible.

We screened the original media report texts from the ARTCDP platform for eligible LAVs-related road traffic crash reports and removed duplicates, after which we extracted and categorised nine key variables for each media-reported crash. All variables were extracted using DeepSeek based on predefined prompts and extraction rules. We evaluated the extraction framework and prompting strategy in a separate methodological study comparing model outputs against manually coded reports and found them to be valid (data not published). The extraction accuracies for the key variables in this study were as follows: time of day (95.8%), type of road (79.4%), location of crash (87.0%), weather conditions (96.8%), province (89.9%), human factors (99.2%), vehicle factors (93.6%), road factors (99.6%) and weather factors (96.8%), suggesting acceptable extraction performance.

Because the level of detail varied across media reports, the denominators differed between analyses according to the availability of information for each variable. As we identified crashes involving LAVs and other motor vehicles from the same media-reporting sources, which may have reduced differential reporting of specific variables between the two groups, we conducted all analyses using available case data for each variable.

### Statistical analysis

We tabulated the number of road traffic crashes involving LAVs and other motor vehicles across the 31 provinces in mainland China to examine patterns across geography, as well as generated bar charts to present the proportions of dangerous driving behaviours, road characteristics, and vehicle conditions. We used chi-squared tests and Fisher’s exact tests, as well as univariate logistic regression models, to compare differences in the characteristics of road traffic crashes involving LAVs *vs*. other motor vehicles. Given the substantial and potentially non-random missingness, we performed further sensitivity analyses and fitted univariate logistic regression models using the full available sample for each variable, and reported the resulting crude odds ratios (ORs) and corresponding 95% confidence intervals (CIs).

We performed statistical analyses in SPSS, version 27(IBM, Armonk, New York, USA) and Microsoft Excel, version 16.102.2 (Microsoft, Redmond, Washington, USA). We considered a two-tailed *P* < 0.05 as the threshold of statistical significance.

## RESULTS

The ARTDCP collected media-reported information about 4,669 crashes involving LAVs and 324,869 crashes involving other motor vehicles in China between January 2015 and August 2025 (**Table 1**). Compared to crashes involving other motor vehicles, those involving LAVs were more frequently reported to occur at nighttime (65.3% *vs*. 29.1%; *P* < 0.001), while less frequently occurred at daytime (34.7% *vs*. 70.9%; *P* < 0.001). As for roadway type of crashes, LAV-related crashes happened more frequently on urban roads (46.9% *vs*. 46.8%; *P* < 0.001) and on expressways (31.9% *vs*. 20.2%; *P* < 0.001) but less frequently on rural roads (4.0% *vs*. 20.0%; *P* < 0.001) than other motor vehicle-related crashes. Besides, crashes involving LAVs were more frequently observed on straight roads without junctions (50.7% *vs*. 29.4%; *P* < 0.001) and bridges (11.6% vs. 9.2%; *P* < 0.001). Conversely, crashes involving other motor vehicles occurred most often at intersections (43.3% *vs*. 23.9%; *P* < 0.001), curved road (6.5% *vs*. 5.1%; *P* < 0.001), tunnels (4.0% *vs*. 3.8%; *P* < 0.001), pedestrian crossing (2.7% *vs*. 1.0%; *P* < 0.001), emergency lane (0.8% *vs*. 0.5%; *P* < 0.001) and bus stops (1.6% *vs*. 0.5%; *P* < 0.001). Based on the 286 reports on crashes involving LAVs and 30,547 reports involving motor vehicles which also contained weather conditions data, road traffic crashes involving LAVs were more likely to occur on rainy days (57.7% *vs*. 53.6%; *P* < 0.001) and sunny days (23.4% *vs*. 12.4%; *P* < 0.001), but less frequently on snow, sleet or hail days (5.9% *vs*. 11.2%; *P* < 0.001) and fog, smog or smoke days (3.2% *vs*. 17.2%; *P* < 0.001) compared to those involving other motor vehicles. The overall distribution patterns of crash characteristics in the complete case sensitivity analyses, which were restricted to reports with complete information for all variables in our analysis, remained generally similar to those observed in the primary analyses (Table S2 in the **Online Supplementary Document**).

**Table 1:** Characteristics of media-reported road traffic crashes involving LAVs *vs*. other motor vehicles in China between January 2015 and August 2025, presented as n (%)*

	Crashes involving LAVs (n = 2,258)	Crashes involving other motor vehicles (n = 179,446)	χ^2^	*P*-value
**Time of day**			738.342	<0.001
Nighttime	773 (65.3)	31,629 (29.1)		
Daytime	411 (34.7)	77,144 (70.9)		
**Type of roadway**			783.507	<0.001
Urban road	1,785 (46.9)	135,618 (46.8)		
Expressway	1,213 (31.9)	58,686 (20.2)		
Rural road	152 (4.0)	57,900 (20.0)		
Other†	654 (17.2)	37,911 (13.1)		
**Location of crash**			589.399	<0.001
Straight road without junction	1119 (50.7)	50,261 (29.4)		
Intersection	528 (23.9)	74,103 (43.3)		
Bridge	256 (11.6)	15,700 (9.2)		
Curved road	113 (5.1)	11,034 (6.5)		
Tunnel	84 (3.8)	6,756 (4.0)		
Pedestrian crossing	21 (1.0)	4,638 (2.7)		
Emergency lane	11 (0.5)	1,310 (0.8)		
Bus stop	10 (0.5)	2,681 (1.6)		
Other‡	67 (3.0)	4,516 (2.6)		
**Weather condition**			76.927	<0.001
Rain	165 (57.7)	16,375 (53.6)		
Sunny	67 (23.4)	3,786 (12.4)		
Snow, sleet, or hail	17 (5.9)	3,410 (11.2)		
Fog, smog, or smoke	9 (3.2)	5,258 (17.2)		
Other§	28 (9.8)	1,718 (5.6)		

ARTCDP – Automated Road Traffic Crash Data Platform, LAV – low-level automated vehicle

*Media-reported road traffic crashes were automatically collected and structured by the ARTCDP. Denominators vary across variables because information was not available for all crashes reported in the media sources, since only news reports that documented the characteristics of the crash were included.

†Includes national and provincial roads, mountain highways, special roads such as in parking garages, and scenic parks

‡Includes toll station, roundabout, *etc*.

§Includes cloudy, extremely low temperature (<0°C) and extremely high temperature (≥37°C).

In logistic regression analyses (Table S3 in the **Online Supplementary Document**), several crash characteristics differed significantly between crashes involving LAVs and those involving other motor vehicles. With daytime and urban roads as reference categories, respectively, nighttime crashes (OR = 4.585; 95% CI = 4.068–5.176) and crashes occurring on expressways (OR = 1.463; 95% CI = 1.357–1.576) were more likely to involve LAVs, while crashes on rural roads were less likely to involve LAVs (OR = 0.171; 95% CI = 0.145–0.202). Using straight roads without junctions as the reference category, crashes occurring at intersections (OR = 0.288; 95% CI = 0.259–0.319), on curved roads (OR = 0.464; 95% CI = 0.379–0.562), and in tunnels (OR = 0.543; 95% CI = 0.430–0.676) were less likely to involve LAVs. Compared with rainy conditions, crashes occurring under snowy, sleet, or hail conditions were less likely to involve LAVs (OR = 0.441; 95% CI = 0.257–0.708).

The number of reported crashes varied greatly across provinces, with Zhejiang, Guangdong, and Shanghai having the highest number of crashes involving LAVs, a pattern that differed somewhat from crashes involving other motor vehicles (Zhejiang, Guangdong, and Sichuan) (**Table 2**). We observed substantial differences in provincial rankings between LAV-related crashes and crashes involving other motor vehicles, particularly in Shanghai, Beijing, and Guangxi. For example, Shanghai ranked 3rd in the number of LAV-related crashes, but 17th in its ranking for crashes involving other motor vehicles.

**Table 2:** Media-reported provincial road traffic crashes involving LAVs *vs*. other motor vehicles by provinces of mainland China between January 2015 and August 2025, presented as n (rank)*

	Crashes involving LAVs (n = 2258)	Crashes involving other motor vehicles (n = 179,446)
**Zhejiang**	611 (1)	24,756 (1)
**Guangdong**	463 (2)	24,201 (2)
**Shanghai**	354 (3)	18,980 (17)
**Jiangsu**	235 (4)	18,889 (4)
**Sichuan**	223 (5)	18,057 (3)
**Henan**	166 (6)	14,933 (6)
**Shandong**	158 (7)	14,632 (8)
**Hubei**	146 (8)	13,765 (12)
**Anhui**	139 (9)	11,622 (9)
**Fujian**	136 (10)	10,762 (11)
**Beijing**	116 (11)	9,445 (21)
**Jiangxi**	104 (12)	9,416 (10)
**Hunan**	99 (13)	9,207 (5)
**Shanxi**	74 (14)	8,379 (16)
**Hainan**	72 (15)	8,119 (22)
**Chongqing**	70 (16)	7,731 (14)
**Shaanxi**	65 (17)	7,393 (18)
**Hebei**	60 (18)	7,262 (13)
**Guangxi**	54 (19)	7,169 (7)
**Guizhou**	28 (20)	5,038 (15)
**Liaoning**	26 (21)	4,920 (20)
**Tianjin**	21 (22)	3,637 (28)
**Yunnan**	21 (23)	3,584 (19)
**Jilin**	16 (24)	2,868 (26)
**Heilongjiang**	14 (25)	2,583 (23)
**Inner Mongolia**	9 (26)	2,010 (27)
**Xinjiang**	5 (27)	1,619 (29)
**Ningxia**	4 (28)	1,615 (24)
**Qinghai**	3 (29)	1,523 (30)
**Tibet**	2 (30)	1,011 (31)
**Gansu**	1 (31)	280 (25)

ARTCDP – Automated Road Traffic Crash Data Platform, LAV – low-level automated vehicle

*Media-reported road traffic crashes were automatically collected and structured by the ARTCDP, which covers the 31 provincial administrative regions of mainland China.

†Only news reports that documented the province of the crash were included.

### Factors related to causing the crashes

**Figure 1:**
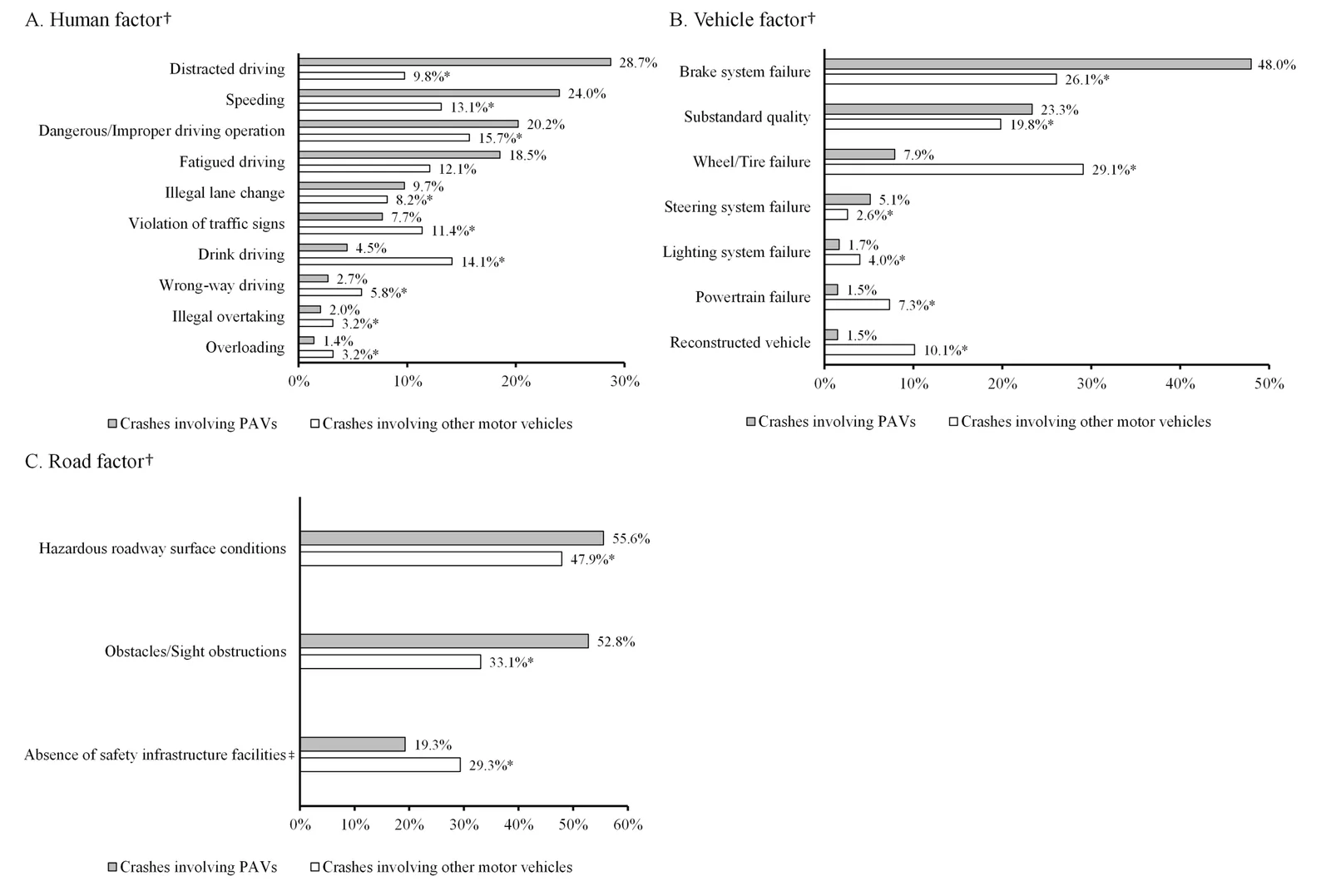
Proportion of contributing factors in media reports on road traffic crashes involving low-level automated vehicles *vs*. other motor vehicles in China between January 2015 and August 2025 due to factors related to humans (**Panel A**), vehicles (**Panel B**), and roads (**Panel C**). Media-reported crashes were automatically collected and structured by the ARTCDP. Denominators varied across variables because information was not available for all variables for each media report. *P < 0.05. †Multiple factors might be involved in a single road traffic crash simultaneously. ‡Road safety infrastructure facilities included comprehensive traffic signals, traffic signs, and road marking lines.

We found differences (*P *< 0.001) between crashes involving LAVs *vs*. other vehicles in terms of whether they had one (79.7% *vs*. 83.7%), two (17.7% *vs*. 14.3%), three (2.4% *vs*. 1.9%), and four or more (0.2% *vs*. 0.1%) contributing factors (**Figure 1**, Panels A–C, **Table 3**). Distracted driving (28.7% *vs*. 9.8%; *P* < 0.05), speeding (24.0% *vs*. 13.1%; *P* < 0.05), dangerous or improper driving operations that were not legally prohibited (20.2% *vs*. 15.7%; *P* < 0.05), fatigued driving (18.5% *vs*. 12.1%; *P* < 0.05) and illegal lane change (9.7% *vs* 8.2%; *P* < 0.05) accounted for a higher proportion of contributing factors to crashes involving LAVs than those involving other motor vehicles.

**Table 3:** Number of contributing factors to media-reported provincial road traffic crashes involving LAVs *vs*. other motor vehicles in mainland China between January 2015 and August 2025, presented as n (%)*

	Crashes involving LAVs (n = 2258)	Crashes involving other motor vehicles (n = 179,446)	χ^2^	*P*-value
**1**	1800 (79.7)	150,188 (83.7)	29.644	<0.001
**2**	399 (17.7)	25,735 (14.3)		
**3**	55 (2.4)	3418 (1.9)		
**≥4†**	4 (0.2)	105 (0.1)		

ARTCDP – Automated Road Traffic Crash Data Platform, LAV – low-level automated vehicle

*Media-reported road traffic crashes were automatically collected and structured by the ARTCDP. Only news reports that documented the cause of the crashes were included.

**†**Cause of road traffic crash was classified into four categories: human factors, vehicle factors, road factors, and other factors.

Brake-related issues (48.0% *vs*. 26.1%; *P* < 0.05), substandard quality of vehicles (23.3% *vs*. 19.8%; *P* < 0.05), and steering system-related issues (5.1% *vs*. 2.6%; *P* < 0.05) contributed to a higher proportion of crashes involving LAVs, which were, in turn, related to a lower proportion of wheel/tire-related issues (7.9% *vs*. 29.1%; *P* < 0.05), reconstructed vehicle factors (1.5% *vs*. 10.1%; *P* < 0.05), powertrain-related issues (1.5% *vs* 7.3%; *P* < 0.05), and lighting system-related issues (1.7% *vs*. 4.0%; *P* < 0.05). Crashes involving LAVs were more likely to occur on roadways with hazardous surface conditions (55.6% *vs*. 47.9%; *P* < 0.05) and those having obstacles or sight obstructions (52.8% *vs*. 33.1%; *P* < 0.05) than crashes involving other motor vehicles, but were less likely occur on roadways without safety infrastructure facilities (19.3% *vs* 29.3%; *P* < 0.05).

## DISCUSSION

Using media-reported data, we explored the characteristics of road traffic crashes involving LAVs and compared them to those involving other motor vehicles. Three key findings emerged: some of the characteristics of LAV-related crashes differed significantly from those involving other motor vehicles; two or more factors were involved in about 20% of crashes involving LAVs; risk factors for crashes involving LAVs differed greatly from those involving other motor vehicles.

Per the media reports, crashes involving LAVs more often occurred on urban roads, expressways, straight road without junctions, and during nighttime. These higher proportions may reflect user behaviour and system use patterns, rather than increased crash risk. For example, these locations typically have good visibility, simple traffic stream, and good road infrastructure. In those situations, as well as long-distance travel during nighttime, drivers tend to be more confident in driver support systems, and LAVs tend to operate at high speed with diminished driver engagement, potentially leading to delayed human driver intervention in unexpected and unusual risk situations [12,13].

Crashes involving LAVs occurred more often in economically developed provinces, likely due to the higher ownership of LAVs in these provinces. Specifically, provinces with higher levels of economic development may see a greater adoption of advanced vehicle technologies. Tesla vehicles, for example, are sold in the highest volumes in Zhejiang, Shanghai, and Jiangsu provinces, which are among the most developed provinces in China [14]. These sales pattern likely contributed to greater LAV use and, consequently, a higher number of LAV-related crashes in these provinces. Further, differences in internet penetration and media infrastructure, which are greater in more developed provinces [15], may lead to crashes being more frequently reported and captured in online news sources. Finally, the larger population and vehicle fleet volume in provinces like Zhejiang, Shanghai, and Jiangsu may mechanically increase the number of observable crash events.

Studies of LAV testing fleets report that human monitoring failures or delayed takeovers are a factor in approximately 30–50% of recorded crashes [16,17], underscoring the continued importance of driver supervision while operating a LAV. However, the magnitude of risky driving behaviours we observed exceeds that reported in most LAV testing studies. For example, distracted driving was documented in 10–15% of LAV testing-related crashes [8], while it accounted for 28.7% of LAV-related crashes in our analysis. Similarly, fatigued driving is rarely reported in testing data sets (<10%), but accounted for 18.5% of LAV-related crashes in our study. These differences may reflect a contrast between behaviours when testing vehicles *vs*. behaviours when operating vehicles in real-world use. Test drivers are professionally trained and mandated to maintain continuous attention, while drivers of LAVs may develop excessive trust in automation and, therefore, engage in secondary tasks (*e.g.* mobile phone use) that impact their safety while driving [8]. It may also reflect reporting biases, with media reports favouring the storytelling aspects of distracted and fatigued drivers.

Existing LAV safety studies predominantly focus on software-related failures, including automated perception and decision-making errors, which together account for approximately 40–60% of reported LAV testing crashes [8,18]. Mechanical failures are reported far less frequently in these datasets, with rates generally below 15% [8,18]. In contrast, we found that 48.0% of LAV-related crashes involved brake-related issues, substantially higher than the 20–30% rate typically reported for both conventional vehicle crashes and LAVs testing fleet crashes [18]. These high rates of brake-related issues may arise from interactions among automated control algorithms, actuators, and human intervention during emergency situations. Delayed or inappropriate handovers between automated and manual control may exacerbate braking-related issues related to LAVs, creating problems such as perceived braking inadequacy, abrupt automated deceleration, emergency braking behaviour, or delayed driver response rather than confirmed mechanical brake failure [19].

Consistent with previous findings [20,21], adverse road and environmental conditions were more frequently associated with LAV-related crashes compared to crashes involving other vehicles. However, unlike previous research reporting the absence of safety infrastructure as a major LAV-related crash determinant [20,21], our results indicate that, compared to crashes involving other vehicles, LAV-related crashes described in media reports were less likely to occur on roadways lacking quality safety facilities (19.3% *vs* 29.3%). This suggests that roadway infrastructure presence alone may be insufficient to ensure safety for LAVs. Instead, crashes may occur more frequently in environments where infrastructure exists, but operational conditions, such as weather, lighting, and visual complexity, challenge both automated systems and human drivers [22,23].

Contrary to Chinese crash statistics suggesting that over 99% of road traffic crashes are attributed both historically and currently to human factors alone [24], our results indicate that 20.8% of crashes involving LAVs reported in the media had two or more contributing factors. We also found that 16.3% of crashes involving other motor vehicles had two or more contributing factors. The discrepancy between our findings and official crash statistics may be due to methodological differences in collecting and counting crash cause statistics. In China, official police-reported crash statistics adopt a single-cause method to report the cause of crash, meaning only one cause of the crash is documented. This practice overlooks the fact that some crashes are caused by multiple factors [25]. Data from systems like ARTCDP may complement existing crash data systems and demonstrate that many crashes may have multiple contributing factors, as described in media reports. Of course, factors derived from media reports should be considered as narrative clues, rather than formally validated causes, and should thus be interpreted cautiously.

### Policy implications

Our findings hold significant policy implications. We identified distinct characteristics relevant to crashes involving LAVs described in media reports that cannot be determined from official crash reports alone. The findings are relevant to policymaking designed to reduce LAV-involved crashes through road safety infrastructure changes, policymaking efforts to legislate LAV manufacturing and sales regulations, and traffic safety laws and enforcement. For example, driver training on the proper use and the limitations of autonomous driving functions should be integrated into national driving license programmes [26]. Further, vehicle manufacturers should improve the usability of human–machine interfaces and the reliability of automated driving software to reduce technical failures (*e.g.* braking system failures and failure to exit automated driving mode). Road authorities should further improve the quality of road infrastructures – such as enhancing lane markings and road surface conditions – to enable LAVs to better perceive road environments. Finally, as recommended by previous research [27,28], a multi-cause method of describing factors that cause crashes should replace the existing single-cause method used to report official crash statistics in China.

### Strengths and limitations

A key strength of this study was our use of online media reports to examine LAV-related crashes in China, a methodological strategy that overcame the lack of available official statistics. We should, however, also acknowledge several limitations. First, media reports are subject to selection bias, since they tend to report more severe and distinct crashes [10], making these data unsuitable for estimating national proportions or detecting temporal trends in LAV-related crashes directly. Limited expertise in road traffic safety among journalists, as well as biases in what readers may want to read in a media report, may have affected the accuracy and depth of reported crash causation. We also acknowledge that some media reports lacked in-depth investigation and that statements from involved parties may be influenced by potential liability concerns (*e.g.* attributing crashes to road or vehicle factors), which could introduce misclassification of causes. Therefore, we reported the results cautiously, with the caveat that all variables are based on media-reported descriptions, rather than official crash investigation reports, and these results should be interpreted as contributing factors, rather than confirmed causal determinants. Second, we extracted crash characteristics from narrative reports rather than official investigations, which may lead to misclassification. Some LAV-related crashes may have been missed if automation-related terms were not explicitly mentioned, and we also failed to determine whether the L1 or L2 technologies caused the crash. Third, substantial missing data may have distorted our findings in a non-random manner, even if crashes involving both types of vehicles were collected from the same media sources with consistent reporting patterns for specific variables. Sensitivity analyses using complete cases produced similar results, but bias from missingness may still have occurred. Finally, exposure data (*e.g.* vehicle kilometres travelled or system usage) were unavailable, preventing estimation of crash risk or causal inference. Therefore, our findings should be interpreted with caution and, where possible, validated through linkage with official police-reported data.

## CONCLUSIONS

As LAVs are becoming increasingly popular, road traffic crashes involving such vehicles have emerged as a public health challenge, both in China and globally. We found that, compared to other vehicles, LAVs were more frequently involved in crashes during nighttime, on expressways, and on straight roads without junctions. In terms of contributing factors, they seemed to be more frequently related to distracted driving, brake-related issues, hazardous road surface conditions, and rainy weather. Policymakers, automobile industry leaders, road engineers, and law enforcement officers should prioritise the prevention of LAV-related crashes, and strategies like improving the quality of road infrastructures and the reliability of software, as well as enhancing driver training about LAVs operation are recommended. Considering potential reporting bias, further research using authoritative data is recommended to testify these findings.

## Additional material


Online Supplementary Document

## Data Availability

**Data availability:** The data that support the findings of this study are available from the ARTCDP upon reasonable request and approval: http://injurybigdata.hpc.csu.edu.cn/product/#/login. The prompts/extraction rules used for the DeepSeek model are available from the corresponding author upon reasonable request.
