# Supplementary material for: Characteristics of media-reported road traffic crashes involving low-level automated vehicles in China, 2015–2025
Source: J Glob Health. 2026 Aug 28;16:04245. doi: 10.7189/jogh.16.04245 (PMC13521493; doi:10.7189/jogh.16.04245)
Supplement: Supplementary file 1 [file jogh-16-04245-s001.pdf]

Supplement to: Zhao M, Yang L, Qi H, Schwebel DC, Xiao W, Wu Y, Cheng P, Hu G. Characteristics of media-reported road traffic crashes involving low-level automated vehicles in China, 2015–2025. J Glob Health. 2026;16:04245.

Table S1. Number of media-reported road traffic crashes involving low-level automated vehicles vs. other motor vehicles in China between January 2015 and August 2025

Category	Chinese search terms	English translation
General automation-related terms	自动驾驶、智能驾驶、辅助驾驶、自动辅助驾驶、自驾驶模式	autopilot, intelligent driving, assisted driving, automated driving, self-driving mode
Longitudinal control functions	自适应巡航、全速自适应巡航、交通感知巡航控制	adaptive cruise control (ACC), full-speed adaptive cruise control, traffic-aware cruise control
Lateral control functions	车道保持辅助、车道居中辅助、车道偏离预警	lane-keeping assistance, lane-centering assistance, lane departure prevention
Combined driver assistance functions	导航辅助驾驶、领航辅助驾驶、高速领航	navigation-assisted driving, Navigate on Autopilot (NOA), highway pilot
Media/platform-specific expressions	智能驾驶模式、车辆自行驾驶、脱手驾驶	autonomous driving mode, vehicle driving itself, hands-free driving
Brand- or system-related terms	特斯拉、小鹏、华为、小米、理想、蔚来	Tesla, XPeng, Huawei, Xiaomi, Li Auto, NIO

Table S2. Comparison of media-reported road traffic crash characteristics involving low-level automated vehicles versus other motor vehicles in China: primary and sensitivity analyses, 2015 and 2025

Characteristics of crash*	Primary analyses‡				Sensitivity analyses‡			
	Crashes involving LAVs†, N (%)	Crashes involving other vehicles, N (%)	χ^2	P	Crashes involving LAVs†, N (%)	Crashes involving other vehicles, N (%)	χ^2	P
Time of day								
Nighttime	773 (65.3%)	31,629 (29.1%)	738.342	<0.001	42 (73.7%)	1,524 (25.0%)	70.349	<0.001
Daytime	411 (34.7%)	77,144 (70.9%)			15 (26.3%)	4,562 (75.0%)		
Type of roadway								
Urban road	1,785 (46.9%	135,618 (46.8%)	745.507	<0.001	35 (61.4%)	1,937 (31.8%)	23.183	<0.001
Expressway	1,213 (31.9%)	58,686 (20.2%)			12 (21.1%)	2,653 (43.6%)		
Rural road	152 (4.0%)	57,900 (20.0%)			5 (8.8%)	805 (13.2%)		
Others§	654 (17.2%)	37,911 (13.1%)			5 (8.8%)	691 (11.4%)		
Location of crash								
Straight road without junction	1,119 (50.7%)	50,261 (29.4%)	589.399	<0.001	28 (49.1%)	2,460 (40.4%)	21.746	0.005
Intersection	528 (23.9%)	74,103 (43.3%)			33 (57.9%)	1,671 (27.5%)		
Bridge	256 (11.6%)	15,700 (9.2%)			5 (8.8%)	623 (10.2%)		
Curved road	113 (5.1%)	11,034 (6.5%)			2 (3.5%)	795 (13.1%)		
Tunnel	84 (3.8%)	6,756 (4.0%)			9 (15.8%)	172 (2.8%)		
Pedestrian crossing	21 (1.0%)	4,638 (2.7%)			1 (1.8%)	114 (1.9%)		
Emergency lane	11 (0.5%)	1,310 (0.8%)			1 (1.8%)	54 (0.9%)		
Bus stop	10 (0.5%)	2,681 (1.6%)			2 (3.5%)	72 (1.2%)		
Others¶	67 (3.0%)	4,516 (2.6%)			1 (1.8%)	125 (2.1%)		
Weather condition								
Rain	165 (57.7%)	16,375 (53.6%)	76.927	<0.001	33 (57.9%)	3,216 (52.8%)	9.806	0.044
Sunny	67 (23.4%)	3,786 (12.4%)			15 (26.3%)	917 (15.1%)		
Snow, sleet or hail	17 (5.9%)	3,410 (11.2%)			3 (5.3%)	605 (9.9%)		
Fog, smog or smoke	19 (3.2%)	5,258 (17.2%)			2 (3.5%)	333 (5.5%)		
Others‖	28 (9.8%)	1,718 (5.6%)			4 (7.0%)	1,015 (16.7%)		

* Media-reported road traffic crashes were automatically collected and structured by the Automated Road Traffic Crash Data Platform (ARTCDP).

† LAVs denotes low-level automated vehicles.

‡ Denominators vary across variables because information was not available for all crashes reported in the media sources since only news reports that documented the characteristics of the crash were included..

§ Other roadway types include national and provincial roads, mountain highways, special roads such as in parking garages and scenic parks

¶ Other locations of crash include toll station, roundabout and so on.

‖ Other weather conditions include cloudy, extremely low temperature (< 0 °C) and extremely high temperature (≥ 37 °C).

Table S3. Logistic regression analysis of characteristics associated with media-reported low-level automated vehicles-related crashes based on media-reported data in China between January 2015 and August 2025

Characteristics of crash*	Odds ratio (95% confidence interval)	*P*
Time of day		
Daytime	Ref	
Nighttime	4.585 (4.068–5.176)	<0.001
Type of roadway		
Urban road	Ref	
Expressway	1.463 (1.357–1.576)	<0.001
Rural road	0.171 (0.145–0.202)	<0.001
Others†	1.113 (1.015–1.219)	0.021
Location of crash		
Straight road without junction	Ref	
Intersection	0.288 (0.259–0.319)	<0.001
Bridge	0.752 (0.653–0.862)	<0.001
Curved road	0.464 (0.379–0.562)	<0.001
Tunnel	0.543 (0.430–0.676)	<0.001
Pedestrian crossing	0.203 (0.127–0.305)	<0.001
Emergency lane	0.336 (0.173–0.583)	<0.001
Bus stop	0.176 (0.088–0.311)	<0.001
Others‡	0.691 (0.533–0.882)	0.004
Weather condition		
Rain	Ref	
Sunny	1.760 (1.308–2.342)	<0.001
Snow, sleet or hail	0.441 (0.257–0.708)	0.001
Fog, smog or smoke	0.579 (0.273–1.075)	0.114
Others§	0.541 (0.356–0.793)	0.003

* Media-reported road traffic crashes were automatically collected and structured by the Automated Road Traffic Crash Data Platform (ARTCDP).

† Other roadway types include national and provincial roads, mountain highways, special roads such as in parking garages and scenic parks

‡ Other locations of crash include toll station, roundabout and so on.

§ Other weather conditions include cloudy, extremely low temperature (< 0 ℃) and extremely high temperature (≥ 37 ℃).
